# Association of glycerolipid metabolism with gut microbiota disturbances in a hamster model of high-fat diet-induced hyperlipidemia

**DOI:** 10.3389/fcimb.2024.1439744

**Published:** 2024-10-04

**Authors:** Lijie Han, Chaowei Hu, Zhiyong Du, Huahui Yu, Yunhui Du, Linyi Li, Fan Li, Yu Wang, Xiaoqian Gao, Xuechun Sun, Zihan Zhang, Yanwen Qin

**Affiliations:** ^1^ Beijing Anzhen Hospital, Capital Medical University, Beijing Institute of Heart Lung and Blood Vessel Disease, Beijing, China; ^2^ The Key Laboratory of Remodeling-Related Cardiovascular Diseases, Ministry of Education, Beijing Anzhen Hospital, Capital Medical University, Beijing, China; ^3^ National Clinical Research Center for Cardiovascular Diseases, Beijing Anzhen Hospital, Capital Medical University, Beijing, China

**Keywords:** gut microbiota, glycerolipid metabolism, high-fat diet, hyperlipidemia, metagenomics, metabolomic analysis

## Abstract

**Background:**

High-fat diet (HFD)-induced hyperlipidemia, which is associated with gut microbiota disturbances, remains a major public health challenge. Glycerolipid metabolism is responsible for lipid synthesis and is thus involved in the development of hyperlipidemia. However, possible association between the HFD-modulated gut microbiome and the glycerolipid metabolism pathway remains unclear.

**Methods:**

Hamsters were fed a HFD for 4 weeks to establish a hyperlipidemia model. Fecal, plasma and liver samples collected from hamsters fed a HFD or a normal chow diet (NCD) were used for integrative metagenomic and untargeted metabolomic analyses to explore changes in the composition and functions of the gut microbiota, and relevant metabolites. Spearman rank correlation analysis was used to explore correlations between gut microbes and circulating glycerolipid metabolites, gut microbes and lipids, and circulating glycerolipid metabolites and lipids.

**Results:**

The gut microbial composition of HFD hamsters showed significant alterations at the phylum, genus, and species levels that were skewed toward metabolic disorders compared with that of NCD hamsters. Functional characterization by KEGG analysis identified enrichment of the glycerolipid metabolism pathway in the gut microbiome of HFD hamsters. Plasma and liver metabolomics further indicated the upregulation and enrichment of glycerolipid metabolites in HFD hamsters. The *Faecalibaculum*, *Allobaculum*, and *Eubacterium* genera were positively correlated with plasma glycerolipid metabolites and lipid indices.

**Conclusion:**

The findings of this study suggest an association between glycerolipid metabolism and the HFD-modulated gut microbiome that is involved in the development of hyperlipidemia.

## 1 Introduction

The remarkable improvement in human living standards has coincided with increased popularity of the high-fat diet (HFD), which facilitates excess energy intake and the development of metabolic syndromes, including hyperlipidemia (Bluher, 2019; Duan et al., 2021; Guo et al., 2022; He et al., 2022). This condition is characterized by an increase in circulating cholesterol, triglyceride, or low-density lipoprotein cholesterol (LDL-C) and serves as a prominent risk factor for atherosclerotic cardiovascular diseases (ASCVD), including coronary artery disease (CAD) and ischemic stroke (Ference et al., 2017; Chew et al., 2023). Accumulating evidence implicates gut microbiota disturbances as one linchpin of HFD-induced hyperlipidemia (Wang and Zhao, 2018; Witkowski et al., 2020). Long-term HFD is associated with fluctuations in the profile and functions of the gut microbiota, which lead to significant changes in relevant metabolites and the development of hyperlipidemia (Ma et al., 2019; Chen et al., 2023). Wan et al. investigated the effects of low-fat diet, moderate-fat diet, and HFD on the human gut microbiota and fecal metabolomics, showing that HFD was associated with decreased abundance of *Faecalibacterium* and increased abundances of *Alistipes* and *Bacteroides* (Wan et al., 2019). Furthermore, metabolites related to ASCVD, encompassing palmitic acid, stearic acid, and arachidonic acid, were also found to be significantly increased in the feces of those on HFD. However, the effects of HFD on the gut microbiota and the mechanisms by which gut microbiota disturbances facilitate hyperlipidemia have not been fully clarified.

Glycerolipids comprise a class of cellular lipids that play important physiological roles in energy storage (primarily in the form of triacylglycerol) and membrane structure (phospholipids and other lipids) (Prentki and Madiraju, 2008). Esterified free fatty acid (FFA) binds to a glycerol backbone to synthesize glycerolipid, which is then hydrolyzed to release non-esterified FFA as part of the glycerolipid/FFA cycle (Prentki and Madiraju, 2008; Prentki et al., 2020). This cycle is primarily responsible for intracellular generation of neutral glycerolipids, including monoacylglycerols (MAGs), diacylglycerols (DAGs), and triacylglycerols, as well as polar glycerolipids (*e.g.*, phospholipids) (Prentki and Madiraju, 2008). It can also produce several intermediate lipids involved in the synthesis of triglyceride, comprising DAGs, lysophosphatidic acid, and phosphatidic acid (Prentki and Madiraju, 2008; Prentki et al., 2020; Farese and Walther, 2023). In this context, previous studies have found an association between glycerolipid metabolism and hyperlipidemia (Prentki et al., 2020; Fu et al., 2022). However, it remains unclear how HFD modulates glycerolipid metabolism. Considering the bridge effect of the gut microbiota on HFD-induced hyperlipidemia, we hypothesize that the modulation of glycerolipid metabolism is associated with gut microbiota disturbances.

In the present study, we utilized integrative metagenomic and metabolomic analyses to investigate the possible association between glycerolipid metabolism and HFD-modulated gut microbiome in the development of hyperlipidemia. Importantly, we employed a hamster model, which exhibits pathophysiological characteristics more closely aligned with those of humans than mice.

## 2 Materials and methods

### 2.1 Animal model

Eighteen male hamsters (age: 12 weeks) supplied by Beijing Huafukang Biotechnology Co. were housed in a specific pathogen-free animal experimental facility at Beijing Anzhen Hospital, Capital Medical University. The hamsters were maintained on a 12:12-hour light/dark cycle and had free access to food and water. The hamsters were randomly divided into two groups: 10 hamsters in the control group were fed a normal chow diet (NCD group) while 8 hamsters in the hyperlipidemia group were fed a HFD of forage containing 21% fat and 0.15% cholesterol (Beijing Huafukang Biotechnology Co.). All experiments were approved by the Key Laboratory of Remodeling-Related Cardiovascular Diseases, were performed in accordance with the guidelines for animal experiments reported by Capital Medical University, and complied with the Guide for the Care and Use of Laboratory Animals published by the US National Institutes of Health (NIH Publication No. 82-23, revised in 1996).

### 2.2 Enzyme-linked immunosorbent assay

After 4 hours of food deprivation (Li et al., 2022), all hamsters were anesthetized with 2% isoflurane (Shandong Ante Animal Husbandry Technology Co. Ltd.) prior to the collection of venous blood samples from the right ventricle. The samples were centrifuged at 3000 rpm for 15 min at 4°C to isolate the supernatant plasma, which was used for ELISAs of the concentrations of circulating lipids, performed in accordance with the kit manufacturer’s instructions (Biosino Bio-Technology and Science Inc.). All lipid values were in the linear range and were calculated based on known protein concentrations.

### 2.3 DNA extraction of fecal samples

Fresh fecal samples collected at the end of the 4-week HFD were snap frozen in liquid nitrogen and stored at −80°C. Fecal DNA was extracted at Novogene Bioinformatics Technology Co., Ltd by using a TIANGEN kit according to the manufacturer’s instructions.

### 2.4 Library construction and metagenomic sequencing

The genomic DNA was randomly sheared into short fragments that were further end repaired, A-tailed, and ligated with the lllumina adapters. Subsequently, PCR was performed attempting to amplify, size select and purify the fragments with adapters to obtain sufficient amplification products for library construction. The library was checked by Qubit2.0 and real-time PCR for quantification and was also bioanalyzer for size distribution detection. Quantified libraries will be pooled and sequenced on Illumina platform, according to effective library concentration and data amount required.

### 2.5 Gene catalogue construction and abundance analysis

We utilized Readfq (V8, https://github.com/cjfields/readfq) to preprocess raw data from the Illumina sequencing platform to obtain clean data for subsequent analysis. In addition, MEGAHIT software (v1.0.4-beta) was further used for metagenome assembly analysis of clean data. Open reading frames (ORF) were predicted using MetaGeneMark and the information with a length less than 100 nt in the prediction results was filtered out. Furthermore, redundancy data were eliminated to obtain the non-redundant initial gene catalogue based on CD-HIT software (V4.5.8). Clean data of each sample was aligned to the initial gene catalogue by using Bowtie2 (Bowtie2.2.4) to calculate the number of reads of the genes, among which genes with reads ≤ 2 were filtered out to finally determine the gene catalogue (Unigenes) for subsequent analysis.

The abundance of each gene in each sample was calculated based on the number of reads aligned and the length of gene, where basic information statistics, core-pan gene analysis, correlation analysis between samples, and Venn diagram analysis of gene number were performed.

### 2.6 Taxonomic and functional annotation

To further define the genes obtained, unigenes sequences were aligned with microbial reference genomes from the Non-Redundant Protein Sequence Database (V20180102) of National Centre for Biological Information by utilizing DIAMOND software (V0.9.9.110) and the alignment results of each sequence with evalue ≤ were selected. LCA algorithm was adopted to determine the final species annotation information of the sequence. Out of the results of LCA annotation and gene abundance table, the abundance of each sample at each taxonomy (kingdom, phylum, class, order, family, genus, or species) and the corresponding gene abundance tables were acquired. Afterwards, ANOSIM analysis (R vegan package, Version 2.15.3) was used to evaluate the differences between groups at each taxonomy level, and the differences were also visualized by abundance clustering heatmap in combination with PCA (R ade4 package, Version 3.2.1). Metastats analysis was used to search for species differences between groups.

For functional annotation, intestinal microbial genes obtained from the DIAMOND sequence aligner were compared with those in the KEGG database (v20180101), where the Best Blast Hit results were selected for subsequent analysis. According to the alignment results, the relative abundance at different functional levels was calculated, as the sum of the relative abundance of genes annotated to the same feature. Differences in metabolic pathways at each taxonomy level were analyzed through linear discriminant analysis effect size (LEfSe) analysis.

### 2.7 Untargeted metabolomic analysis of plasma and liver samples

All samples were analyzed using a Vanquish UHPLC system (ThermoFisher, Germany) coupled with an Orbitrap Q ExactiveTMHF mass spectrometer. We performed peak alignment, peak picking, and quantitation using the Compound Discoverer 3.1 software (CD3.1, ThermoFisher). These peaks were matched with the mzCloud (https://www.mzcloud.org/), mzVault and MassList database to obtain qualitative and relative quantitative results of metabolites, which were annotated to the KEGG database (https://www.genome.jp/kegg/pathway.html).

### 2.8 Statistical analysis

Plasma lipid data (continuous variables) are presented as means ± standard error of the mean (SEM) and were compared using Student’s *t* test. Non-normally distributed gut metagenomic data were analyzed using the Wilcoxon rank sum test. Taxonomy assignment, functional profiling, and microbial metabolic pathway reconstruction were performed from clean data using HUMAnN2 software. The heatmaps of metagenomic data were generated by R software (v4.1.2). Spearman rank correlation analysis was used to explore associations between the following: gut microbes and circulating glycerolipid metabolites, gut microbes and lipids, and circulating glycerolipid metabolites and lipids.

Functional characterization of the metabolites was analyzed by R software. The inter-group comparison of the metabolite levels was conducted using a Wilcoxon rank sum test, with a *P* value < 0.05 considered to indicate statistical significance. Differentially changed metabolites were annotated using the Kyoto Encyclopedia of Genes and Genomes (KEGG) database (http://www.kegg.jp/kegg/compound/), using a *P* value cutoff of < 0.1.

## 3 Results

### 3.1 Hyperlipidemia and decrease in gut microbiota genes induced by HFD

**Figure 1:**
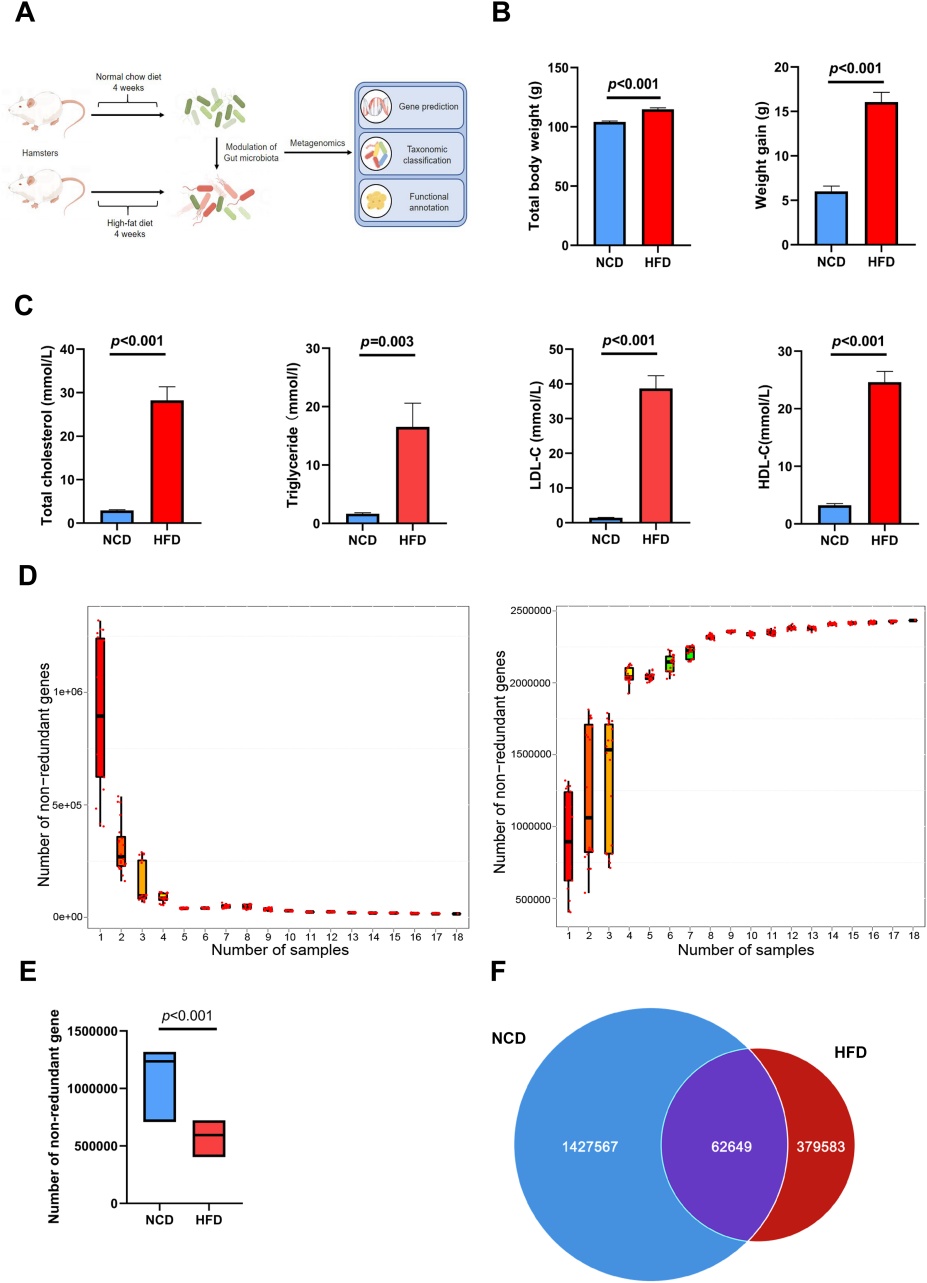
Altered gut microbial genes in high-fat diet (HFD)-induced hyperlipidemia hamsters. **(A)** Experimental protocols of HFD modeling and metagenomic sequencing **(B)** After feeding HFD for 4 weeks, total body weight and the increment of body weight in HFD group were both significantly higher than those in NCD group; **(C)** Circulating lipids were increased in hamsters fed HFD; **(D)** The core-pan rarefaction curves gradually approached saturation with the increase of sample size, suggesting a high-quality sequencing data; The box plot **(E)** and Venn plot **(F)** showed that the number of non-redundant genes of gut microbiota in HFD hamsters was significantly decreased compared with that in normal chow diet (NCD) hamsters.

**Figure 1A** illustrates the procedures used for HFD modeling and metagenomic analysis. After feeding HFD for 4 weeks, the increment of body weight and total body weight in HFD group were both significantly higher than those in NCD group (weight gain: 16.07 ± 1.10 g vs. 5.81 ± 0.60 g, p < 0.001; total body weight: 114.87 ± 1.14 g vs. 104.71 ± 0.68 g, P < 0.001; **Figure 1B**). Moreover, lipid indices, including circulating levels of cholesterol, triglyceride, LDL-C, and high-density lipoprotein-cholesterol (HDL-C), were increased in hamsters fed a 4-week HFD compared with those in NCD hamsters, validating the establishment of hyperlipidemia (**Figure 1C**).

Fecal DNA was extracted from stool samples, sequenced on the Illumina platform, and a total of 113 Gb 125-bp paired-end reads were generated, with an average of 6294 ± 96.01 (s.e.) million reads per sample. The core-pan rarefaction curves gradually approached saturation with the increase in sample number, suggesting that there were sufficient sequencing data to reflect the composition of the gut microbiota (**Figure 1D**). The gene abundance in each sample exhibited an intra-group correlation with that in any other sample in both HFD and NCD hamsters (**Supplementary Figure S1**). Notably, the number of non-redundant genes in the gut microbiota of HFD hamsters was significantly lower than that of NCD hamsters (**Figures 1E, F**), consistent with previous studies (Cotillard et al., 2013; Le Chatelier et al., 2013; Liu et al., 2017).

### 3.2 HFD-associated alterations in gut microbial composition at different taxonomic levels

**Figure 2:**
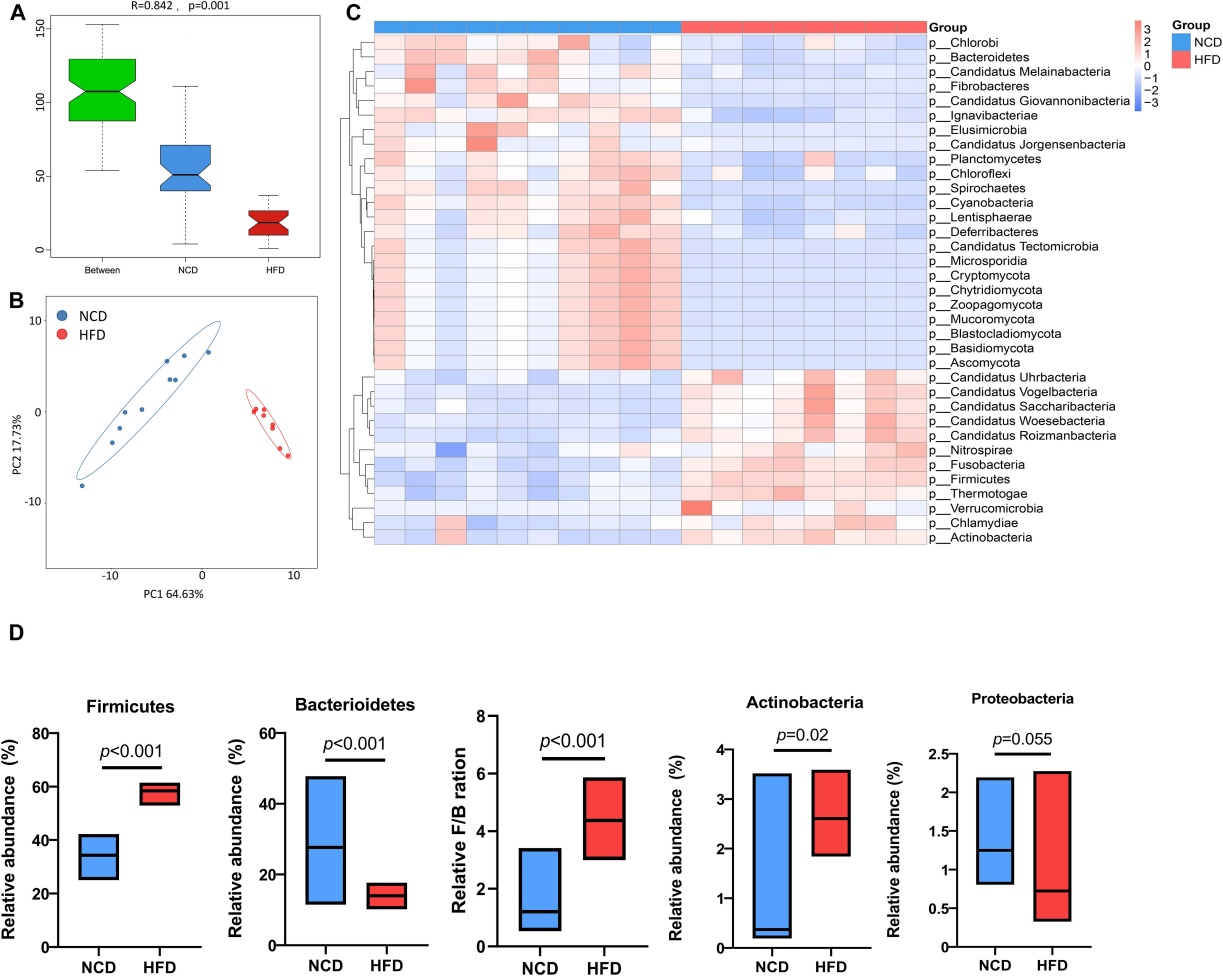
High-fat diet-associated alterations in gut microbial composition at the phylum level. The analysis of similarity via ANOSIM **(A)** and the principal coordinate analysis (PCoA) based on Bray–Curtis dissimilarity **(B)** both revealed a remarkable difference in the composition of gut microbiota at the phylum level between high-fat diet (HFD) and normal chow diet (NCD) hamsters. **(C)** The heat map illustrated top 35 differential phyla between HFD and NCD hamsters. **(D)** The relative abundances of four phyla that dominate in gut microbiota were changed, among which *Firmicutes* and *Actinobacteria* were increased, and *Bacteroidetes* was decreased, resulting in a higher *Firmicutes/Bacteroidetes* (F/B) ratio under HFD (calculated by *firmicute* divided by *bacteroidetess*).

To further investigate specific effects exerted by HFD on gut microbial composition, the differentially expressed genes were annotated to the corresponding bacterial taxa, including phyla, genera, and species. Analysis of similarities (ANOSIM) of microbes and principal coordinate analysis (PCoA) based on Bray–Curtis dissimilarity synergistically revealed a significant separation of microbial communities between HFD and NCD hamsters (**Figures 2A, B**). Taxonomic annotation and abundance profiling of the gut microbiota at the phylum level further showed altered composition of the gut microbiota in HFD hamsters (**Figure 2C**; **Supplementary Table S1**). In particular, the relative abundances of four phyla were changed, among which Firmicutes and Actinobacteria were increased, and Bacteroidetes was decreased, resulting in a higher Firmicutes/Bacteroidetes (F/B) ratio under HFD, which is associated with metabolic disorders (Machate et al., 2020). The Proteobacteria phylum also showed a propensity to increase under HFD (**Figure 2D**).

**Figure 3:**
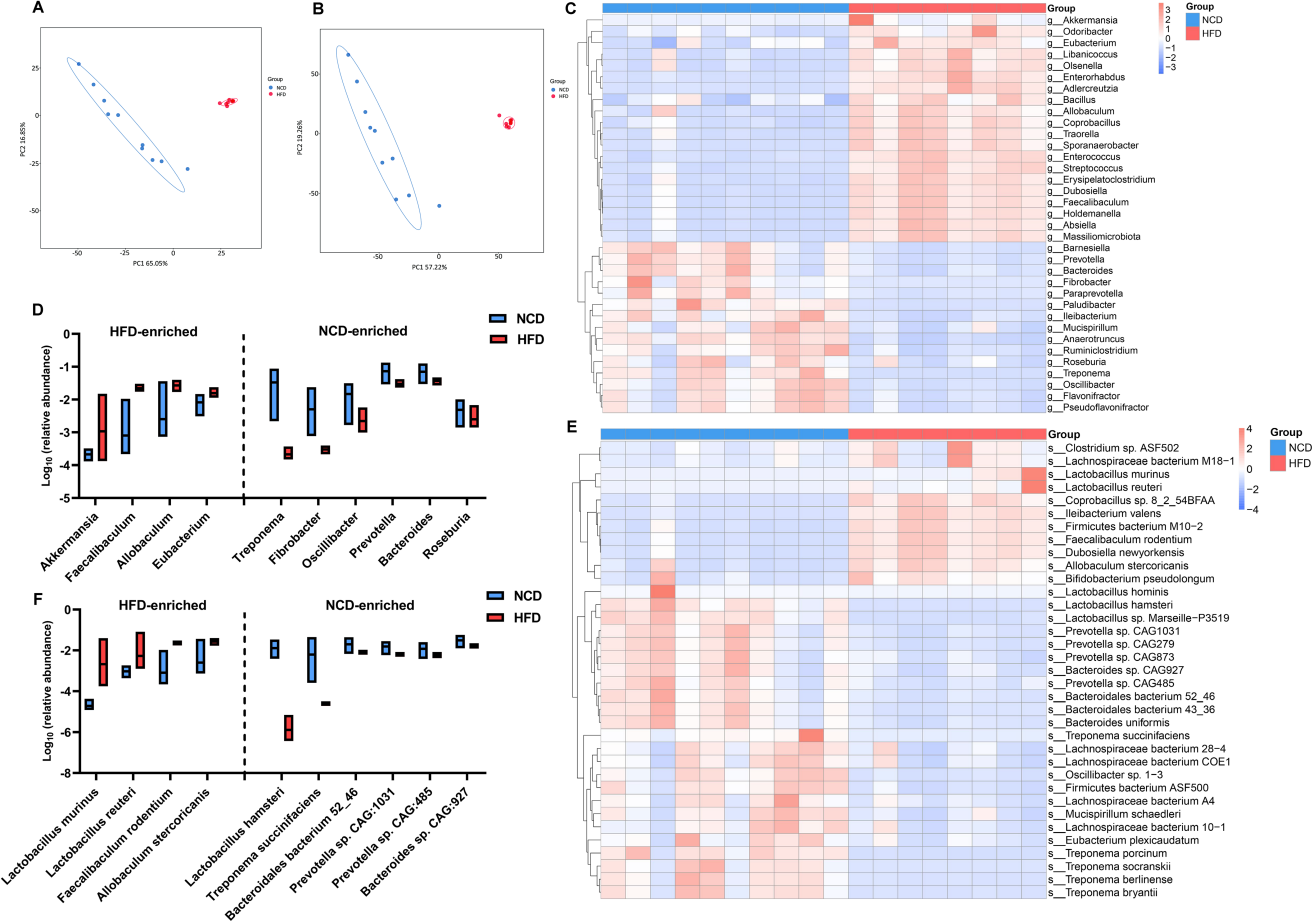
High-fat diet-associated alterations in gut microbial composition at the genus and species levels. The principle component analysis (PCA) based on the features of the gut microbiota showed a remarkable spatial separation of gut microbial composition at both genus **(A)** and species levels **(B)** between high-fat diet (HFD) and normal chow diet (NCD) hamsters. The heat map illustrated 35 most abundant and statitiscally significant genera **(C)** and species **(D)** between HFD and NCD hamsters, among which the top 10 genera **(E)** and species **(F)** were exhibited in the box plots.

As expected, there were also statistically significant differences in the communities at the genus and species levels (**Supplementary Figures S2A, B**). Principle component analysis (PCA) based on the features of the gut microbiota further substantiated the spatial separation of communities at both levels (**Figures 3A, B**). Among the 35 most differential genera (**Figure 3C**; **Suplementary Table S2**), the 10 most abundant were further illustrated using a box plot (**Figure 3D**). Notably, *Allobaculum*, *Faecalibaculum*, and *Eubacterium*, which belong to the Firmicutes phylum, were significant overrepresented in HFD hamsters. Similarly, **Figure 3E** and **Supplementary Table S3** illustrates the top 35 differentially enriched species. Among the 10 most abundant species (**Figure 3F**), *Allobaculum stercoricanis*, *Faecalibaculum rodentium*, *Lactobacillus murinus*, and *Lactobacillus reuteri* showed increased abundances in HFD hamsters, in contrast to *Lactobacillus hamster*, *Treponema succinifacisen*, *Bacteroidales bacterium* 52_46, *Prevotella* sp. CAG1031, *Prevotella* sp. CAG485, and *Bacteroides* sp. CAG927, which showed decreased abundances.

### 3.3 Association between the HFD-modulated gut microbiome and glycerolipid metabolism

**Figure 4:**
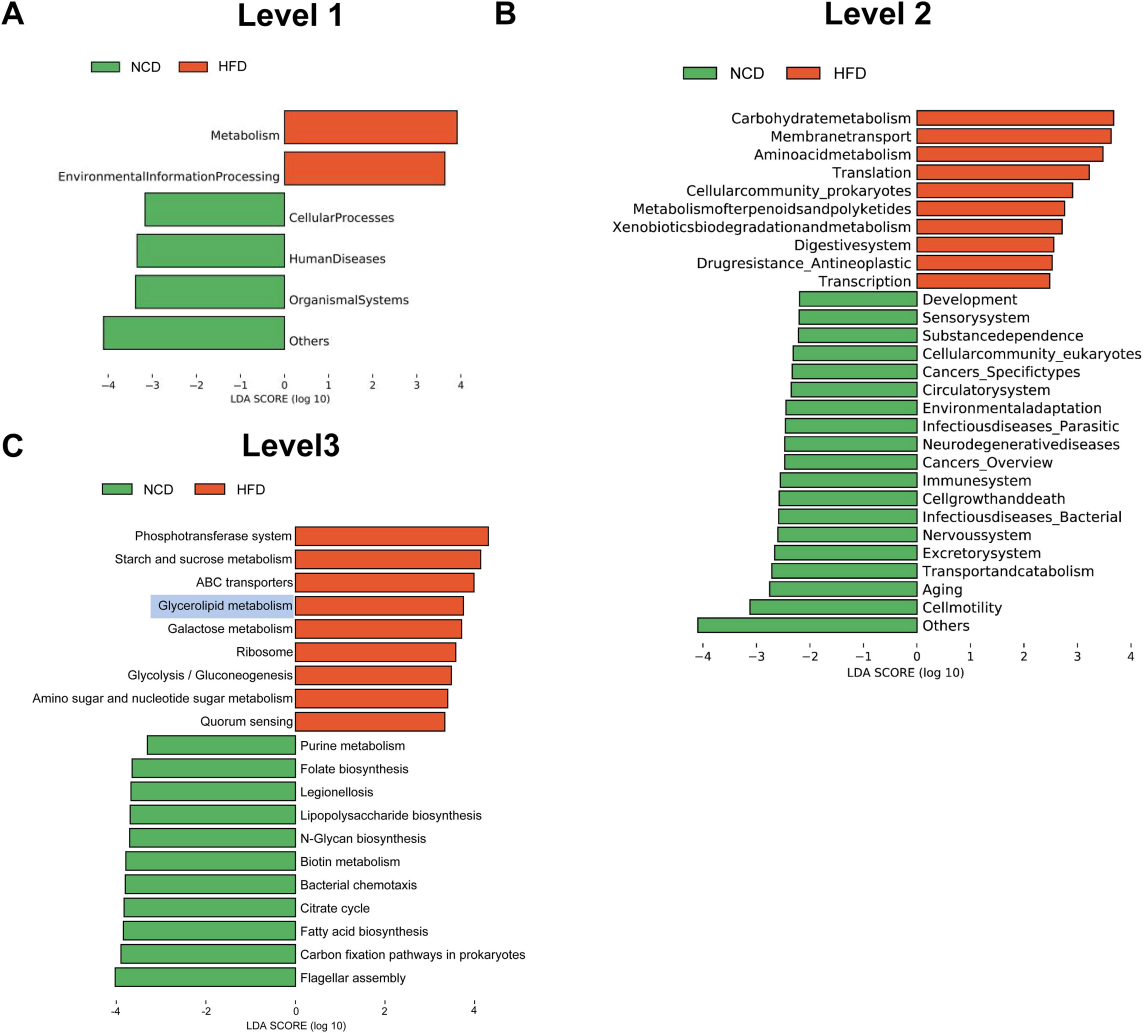
Functional characterization of the differentially enriched microbiome. LEfSe analysis exhibited Kyoto Encyclopedia of Genes and Genomes (KEGG) pathways differentially enriched (*P*<0.05, log_10_(linear discriminant analysis (LDA) score) >2) in high-fat diet (HFD) or chow diet (NCD) hamsters stratified by Level 1 **(A)**, Level 2 **(B)** and Level 3 **(C)**.

Next, the functional profiles of the differentially expressed gut microbial genes between HFD and NCD hamsters were characterized by KEGG enrichment analysis. Specifically, KEGG orthologues that were differentially enriched (cutoffs: *P* < 0.05 and log_10_(linear discriminant analysis (LDA) score) > 2) in the HFD or NCD groups at various levels (Levels 1–3) were subjected to LEfSe analysis (**Figure 4**). At Level 1, we observed enrichment of pathways related to metabolism and environmental information processing in HFD hamsters (**Figure 4A**). At Level 2, 29 significantly different pathways between HFD and NCD hamsters were identified, with 10 pathways enriched in HFD hamsters, including numerous metabolic pathways (*e.g.*, carbohydrate metabolism and amino acid metabolism) (**Figure 4B**). The top 10 pathways at Level 3 in each group are illustrated in **Figure 4C**, notably revealing that glycerolipid metabolism was enriched in HFD hamsters with a relatively great abundance of log_10_(LDA score)=3.80 (*P*=0.004). This finding demonstrated an association between HFD-induced gut microbial disturbances and alterations in the glycerolipid metabolism pathway.

### 3.4 HFD-mediated upregulation of glycerolipid metabolites

**Figure 5:**
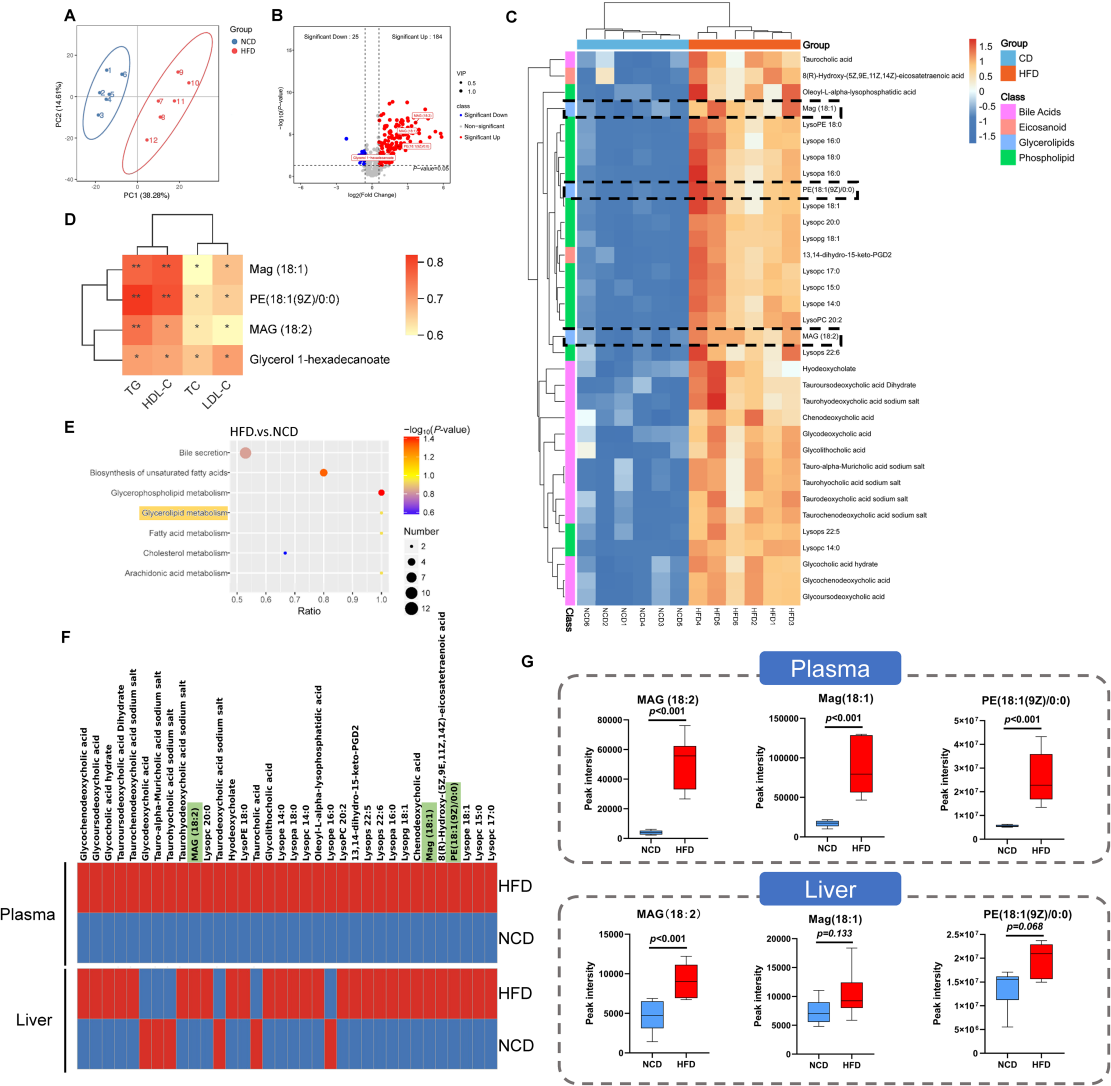
Identification of related metabolites in plasma by untargeted metabolomic analysis in hamsters with high-fat diet and normal chow diet. **(A)** The principle component analysis (PCA) showed that the metabolic spectrums in high-fat diet (HFD) and normal chow diet (NCD) hamsters were completely separated; **(B)** The volcano plot showed 209 metabolites altered in HFD hamsters compared with NCD hamsters, among which 184 metabolites (VIP>1; *P*<0.05; Fold change>1.5) and 4 glycerolipid metabolites were significantly upregulated; **(C)** The heat map illustrated the 34 most differentially changed metabolites associated with hyperlipidemia (VIP>1; P<0.05; |log2(Fold change)|>2; involved in hyperlipidemia development). The black dotted frames highlighted glycerolipid metabolites; **(D)** Heat map of Spearman’s rank correlation coefficients between 4 glycerolipid metabolites and circulating lipids. **(E)** The Kyoto Encyclopedia of Genes and Genomes (KEGG) analysis identified functional pathways enriched in HFD hamsters. **(F)** The heat maps showed the 34 metabolites associated with hyperlipidemia in plasma and liver tissue. **(G)** The box plots showed that glycerolipid metabolites, including Mag (18:1), MAG (18:2) and PE (18:1(9Z/0:0)), were increased in HFD hamsters compared with those in NCD hamsters. *P < 0.05; **P < 0.01.

Considering the enrichment of metabolism-related pathways suggested by the KEGG analysis, we further investigated the plasma metabolic profiles of HFD and NCD hamsters using untargeted metabolomics analysis. The heat map shown in **Supplementary Figure S3** illustrates all of the significantly changed metabolites. As expected, the metabolic spectrums of the two groups were completely separated (**Figure 5A**). After rigorous screening (screening criteria: variable importance in projection [VIP] score > 1; *P* < 0.05; |Fold change| > 1.5), these significantly changed metabolites show enrichment of 184 metabolites in HFD hamsters and 25 metabolites in NCD hamsters (**Figure 5B**). Consistent with the enrichment of glycerolipid metabolism found in the gut microbiota, we also identified four upregulated glycerolipid metabolites in the plasma of HFD hamsters (**Figure 5B**): MAG (18:2), MAG (18:1), phosphatidylethanolamine PE (18:1(9Z/0:0) and glycerol 1-hexadecanoate. We further screened top 34 significantly changed metabolites included in the metabolite classes that were associated with the development of hyperlipidemia (*e.g.*, bile acids) (criteria: VIP > 1; *P* < 0.05; |log_2_(Fold change) | > 2). Notably, MAG (18:2), MAG (18:1), and PE (18:1(9Z/0:0)) were among these metabolites (**Figure 5C**). Furthermore, glycerolipid metabolites were positively correlated with circulating lipids (all *P* < 0.05), indicating an association between glycerolipid metabolism and hyperlipidemia (**Figure 5D**). KEGG analysis further supported the functional enrichment of glycerolipid metabolism, in addition to bile secretion, glycerophospholipid metabolism, and other metabolic pathways (**Figure 5E**). Moreover, the untargeted metabolomics in liver tissue indicated a consistency of glycerolipid metabolites with those in plasma (**Figure 5F**), where MAG (18:2) was significantly increased, while Mag (18:1) and PE (18:1(9Z/0:0)) also showed an upregulated trend (**Figure 5G**).

### 3.5 Correlation analysis of the gut microbiota, glycerolipid metabolites, and circulating lipids

**Figure 6:**
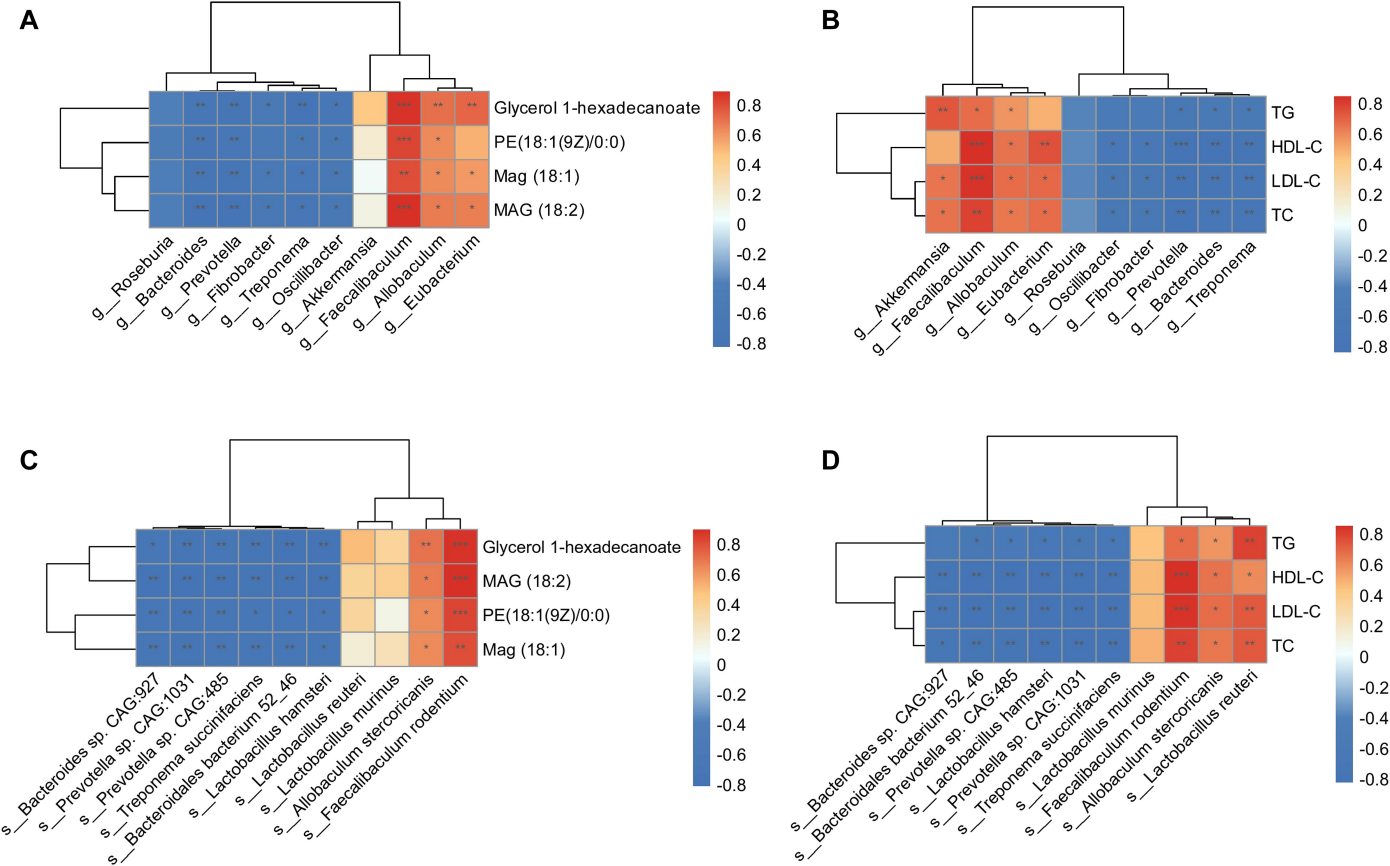
Correlation analysis of gut microbiota, glycerolipid metabolites, and circulating lipids. Heat maps of Spearman’s rank correlation coefficients between 4 glycerolipid metabolites and 10 differentially abundant gut microbial genera **(A)**, circulating lipids and 10 differentially abundant gut microbial genera **(B)**, 4 glycerolipid metabolites and 10 differentially abundant gut microbial species **(C)**, as well as circulating lipids and 10 differentially abundant gut microbial species **(D)**. *P < 0.05; **P < 0.01; ***P < 0.001.

Regarding the altered composition of the gut microbiota and functional enrichment of glycerolipid metabolism in HFD hamsters, we conducted Spearman’s rank correlation analyses between gut microbes and circulating glycerolipid metabolites, gut microbes and lipids, and circulating glycerolipid metabolites and lipids. The 10 most abundant bacterial genera and species (**Figure 3**) and the four upregulated glycerolipid metabolites in HFD hamsters were pooled for this analysis. *Faecalibaculum* showed strong positive correlations with all four glycerolipid metabolites, while *Eubacterium* and *Allobaculum* were correlated with MAG (18:1), MAG (18:2), and glycerol 1-hexadecanoate (**Figure 6A**). The correlation analysis between gut microbes and lipid indices further substantiated the associations between these three bacterial genera and circulating lipids (**Figure 6B**). Conversely, the *Treponema*, *Fibrobacter*, *Bacteroides*, *Prevotella*, and *Oscillibacter* genera all showed negative correlations with glycerolipid metabolites and circulating lipids (**Figures 6A, B**). Notably, *Akkermansia* was also positively correlated with circulating lipids, but not with glycerolipid metabolites, indicating that *Akkermansia* may be involved in hyperlipidemia via other metabolic pathways (**Figure 6A**). These data suggested potential roles of *Faecalibaculum*, *Eubacterium*, and *Allobaculum* in glycerolipid metabolism and the development of hyperlipidemia.

Further investigation of these correlations at the species level showed that *Faecalibaculum rodentium* and *Allobaculum stercoricanis* were significantly associated with glycerolipid metabolites and circulating lipids (**Figures 6C, D**). In contrast, species pertaining to the *Treponema*, *Fibrobacter*, *Bacteroides*, *Prevotella*, and *Oscillibacter* genera showed negative correlations with these indices. Taken together, the correlation analyses identified specific genera and corresponding species that were associated with glycerolipid metabolism in HFD hamsters, resulting in lipid synthesis and hyperlipidemia.

## 4 Discussion

**Figure 7:**
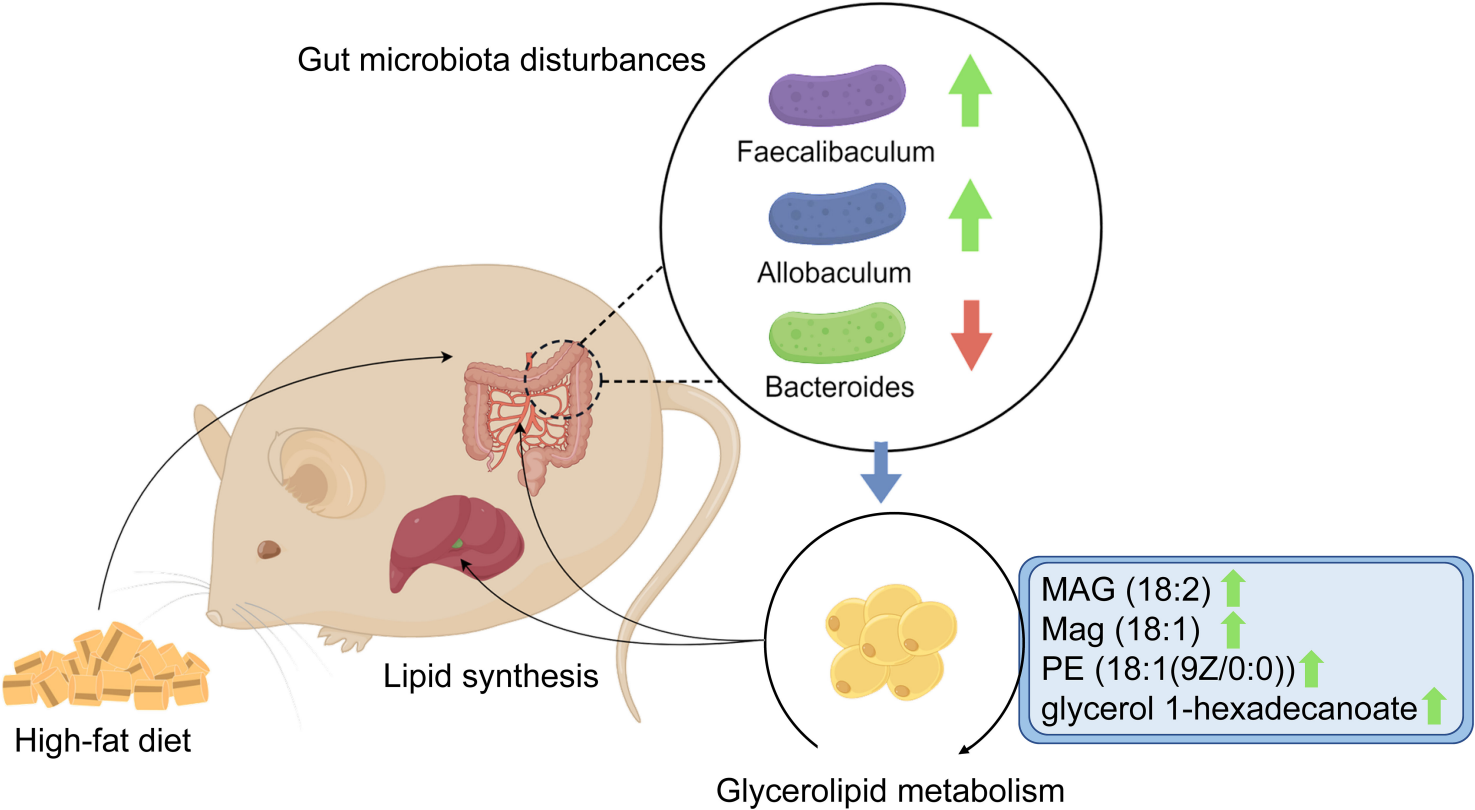
Graphical abstract. The schematic diagram shows the potential association between glycerolipid metabolism and HFD-induced gut microbiota disturbances, which is found to be involved in hyperlipidemia in a hamster model.

To the best of our knowledge, this research is the first to focus on the association between glycerolipid metabolism and HFD-induced gut microbiota disturbances, which was found to be involved in hyperlipidemia in a hamster model. Using integrative fecal metagenomic and plasma metabolomic analyses, we observed that HFD significantly altered the gut microbial composition at the phylum, genus, and species levels. Functional characterization identified an enriched glycerolipid metabolism pathway in the HFD-modulated gut microbiome. Furthermore, glycerolipid metabolites were also upregulated in the plasma and liver tissue. Three genera showed positive correlations with circulating glycerolipid metabolites and lipid indices: *Faecalibaculum*, *Allobaculum*, and *Eubacterium* (**Figure 7**). The results of our research provide a novel mechanistic insight into HFD-induced hyperlipidemia that may lead to improvements in the prevention and treatment of hyperlipidemia.

Hyperlipidemia remains a major public health challenge on a global scale, and is often insufficiently managed, treated, and controlled (Fox et al., 2018; Gong et al., 2021; Lu et al., 2021). Data from the Global Burden of Diseases 2019 revealed that, although the annual mortality among patients with hyperlipidemia showed a decreased propensity from 2000 to 2019, over 4 million patients nevertheless succumbed to the condition in 2019 (Chew et al., 2023). It is also notable that annual mortality even increased in certain low-income regions, such as Southeast Asia. The China-PEACE project indicated that 33.8% of middle-aged and elderly Chinese people had hyperlipidemia. More surprisingly, only 17.1% of patients with established ASCVD received lipid-lowering treatment, with an LDL-C goal (≤ 70 mg/dL) attainment rate of 44.8% (Lu et al., 2021). These data collectively emphasize a dilemma in the prevention and therapy of hyperlipidemia. Given that the factors contributing to the suboptimal control of hyperlipidemia are obscure and intricate, it is reasonable to speculate that residual disease mechanisms exist that current lipid-lowering medications may not fully address. A plethora of studies have attributed high-fat and high-energy diets as the main causative factors in hyperlipidemia, being responsible for the imbalance of energy supply and consumption (Pereira et al., 2005). Therefore, the inhibition of lipid synthesis holds promise for the prevention of hyperlipidemia development.

Accumulating studies have demonstrated that a HFD can result in hyperlipidemia by modulating the composition and function of the gut microbiota (Huang et al., 2019; Qiao et al., 2022); however, the mechanisms remain somewhat unclear. Chang et al. found that HFD led to increased abundance of Firmicutes and decreased abundance of Bacteroidetes, followed by an augmentation of the F/B ratio (Chang et al., 2015). This observation was validated in our research, suggesting the robustness of HFD model establishment. The increased abundance of Firmicutes in the host increases the absorption and ease of conversion of dietary calories into fat, which can accumulate in the circulation and thereby promote the development of hyperlipidemia (Meijnikman et al., 2020). Firmicutes and Bacteroidetes can remain stable independent of the duration of HFD (Chen et al., 2023), demonstrating a long-term association between gut microbiota-related metabolic disorders and hyperlipidemia. On the other hand, this persistency could be partially explained by the irreversible impact of HFD on gut microbiota. Sonnenburg et al (Sonnenburg et al., 2016). found that when mice were switched from the 6-week high-microbiota-accessible carbohydrates (MAC) diet to the 7-week low-MAC diet (namely, western diet), 60% of taxa decreased in abundance at least fourfold. Intriguingly, approximately half of the decreased taxa did not remarkably restore when returning to high-MAC diet. In addition, taxa driven to low abundance when dietary MACs were scarce were inefficiently transferred to the next generation, and were at increased risk of becoming extinct within an isolated population. Taken together, these results indicate a paramount important of the interventions on gut microbiota and may be incorporated in the therapy of hyperlipidemia.

In this study employing a pathophysiologically appropriate hamster model for the exploration of dietary-induced hyperlipidemia, we also identified *Faecalibaculum* and *Eubacterium*, two genera of the Firmicutes phylum, as being positively correlated with circulating cholesterol, triglyceride, and LDL-C. Previous studies revealed that increased abundance of intestinal *Faecalibaculum* in HFD-induced obesity mice was associated with elevated serum levels of cholic acid, chenodeoxycholic acid, and deoxycholic acid, reduced bile acid synthesis, and increased cholesterol accumulation via the hepatic farnesoid X receptor-small heterodimer partner axis (Xu et al., 2023). *Faecalibaculum* has also been positively correlated with the inflammatory response that may be involved in the development of ASCVD (Chen et al., 2012). In the present study, we also observed significant correlations between *Faecalibaculum* and upregulated glycerolipid metabolites, namely MAG (18:2), MAG (18:1), PE (18:1(9Z/0:0)), and glycerol 1-hexadecanoate. Furthermore, there was functional enrichment of glycerolipid metabolism in the gut microbiota of HFD hamsters. Given that functional annotation analyses are predictive in nature, further research is required to investigate the association between *Faecalibaculum* and glycerolipid metabolism and the underlying mechanisms. It is also notable that, although many gut microbial-related metabolites involved in hyperlipidemia have been identified, such as short-chain fatty acids, secondary bile acids, glutamic acid, indole and trimethylamine N-oxide (Jia et al., 2021; Vourakis et al., 2021), there is only sporadic evidence to associate hyperlipidemia with the modulation of glycerolipid metabolism by disturbances in the gut microbiota. It should be notable that only male hamsters were utilized in the current study primarily due to the sexual difference in the response of lipid metabolism and gut microbiota to HFD that the microbial diversity decreases in male mice fed a HFD but not in female mice fed a HFD (Zhu et al., 2023), suggesting a resistance of female mice to hyperlipidemia. This phenomenon could be explained by the significant increase of hyperlipidemia-related microbiome in male mice, in contrast to the enrichment of *Akkermansia* in female mice that protects against the development of hyperlipidemia. However, the sexual difference in gut microbiome associated with hyperlipidemia still requires further research.

The glycerolipids include numerous cellular lipids that are involved in the glycerolipid/FFA cycle. This cycle produces several intermediate lipids responsible for the synthesis of triglyceride, including lysophosphatidic acid, phosphatidic acid, and DAG (Prentki and Madiraju, 2008), demonstrating the importance of glycerolipid metabolism in the development of hyperlipidemia, of which hypertriglyceridemia is a major type and is associated with the progression of ASCVD (Sandesara et al., 2019; Laufs et al., 2020). Furthermore, He et al. found that alleviation of hyperlipidemia was concomitant with downregulated expression of glycerolipid metabolism-related genes, including *Lpin1*, *Hadha*, *Aldh3a2*, *Acox1*, *Elovl3*, *Elovol5*, and *Agpat3* (He et al., 2022). Accordingly, we observed positive correlations between glycerolipid metabolites and circulating lipids. In addition, glycerolipid metabolites were positively associated with *Faecalibaculum*, *Allobaculum*, and *Eubacterium*. Taken together, we hypothesize that HFD may induce the enrichment of glycerolipid metabolism via gut microbial disturbances that are followed by lipid synthesis and supervening hyperlipidemia. Our findings may lead to novel targets that alleviate the sluggishness of hyperlipidemia. Potential therapeutic modalities for hyperlipidemia may incorporate probiotics and dietary patterns (Nyangale et al., 2012; Jia et al., 2023) to create a healthy and stable intestinal microenvironment by modulating the composition of bacteria and promoting favorable metabolic effects, with inhibition of the corresponding metabolites (Jia et al., 2021). However, such approaches require further investigation, including in-depth research on the mechanisms linking the gut microbiota and glycerolipid metabolic patterns, as well as the signaling pathways and cellular targets of glycerolipid metabolites.

## 5 Limitations

Despite the robustness of our findings, there are several limitations in our study. First, a hamster model used in our study may not fully reflect the effects of a HFD on the human gut microbiota and metabolism, requiring further research to validate the association between glycerolipid metabolism and gut microbiota disturbances based on human fecal samples. Second, although we observed a significant correlation between several specific bacteria and glycerolipid metabolites, in-depth experimental studies, *e.g.*, fecal microbiota transplantation and targeted interventions of metabolic pathways, are required to confirm the important role of gut microbiota in glycerolipid metabolism. Finally, only male hamsters were utilized in the current study, thus potential impact resulting from the sexual difference should not be neglected in further studies.

## Data Availability

The datasets presented in this study are available in online repositories and can be found with the accession number: NCBI, PRJNA1163029.
