# Supplementary material for: Association of glycerolipid metabolism with gut microbiota disturbances in a hamster model of high-fat diet-induced hyperlipidemia
Source: Front Cell Infect Microbiol. 2024 Oct 4;14:1439744. doi: 10.3389/fcimb.2024.1439744 (PMC11486926; doi:10.3389/fcimb.2024.1439744)
Supplement: Supplementary file 1 [file DataSheet1.docx]

Supplementary Material

## Supplementary Figures

**
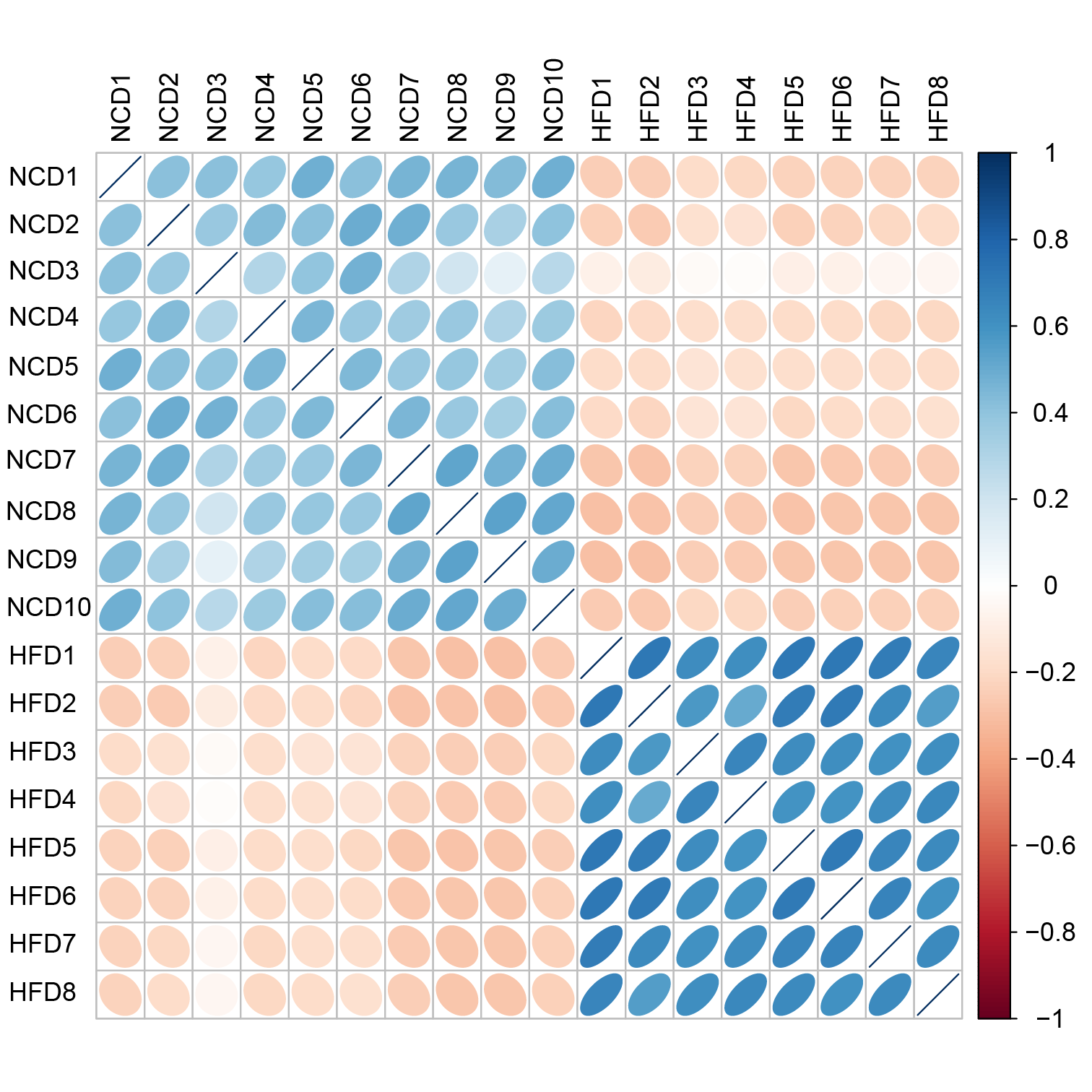
**

**Supplemental figure 1.** Correlation of the gene abundance in each sample with any other sample

**A**

**B**

**
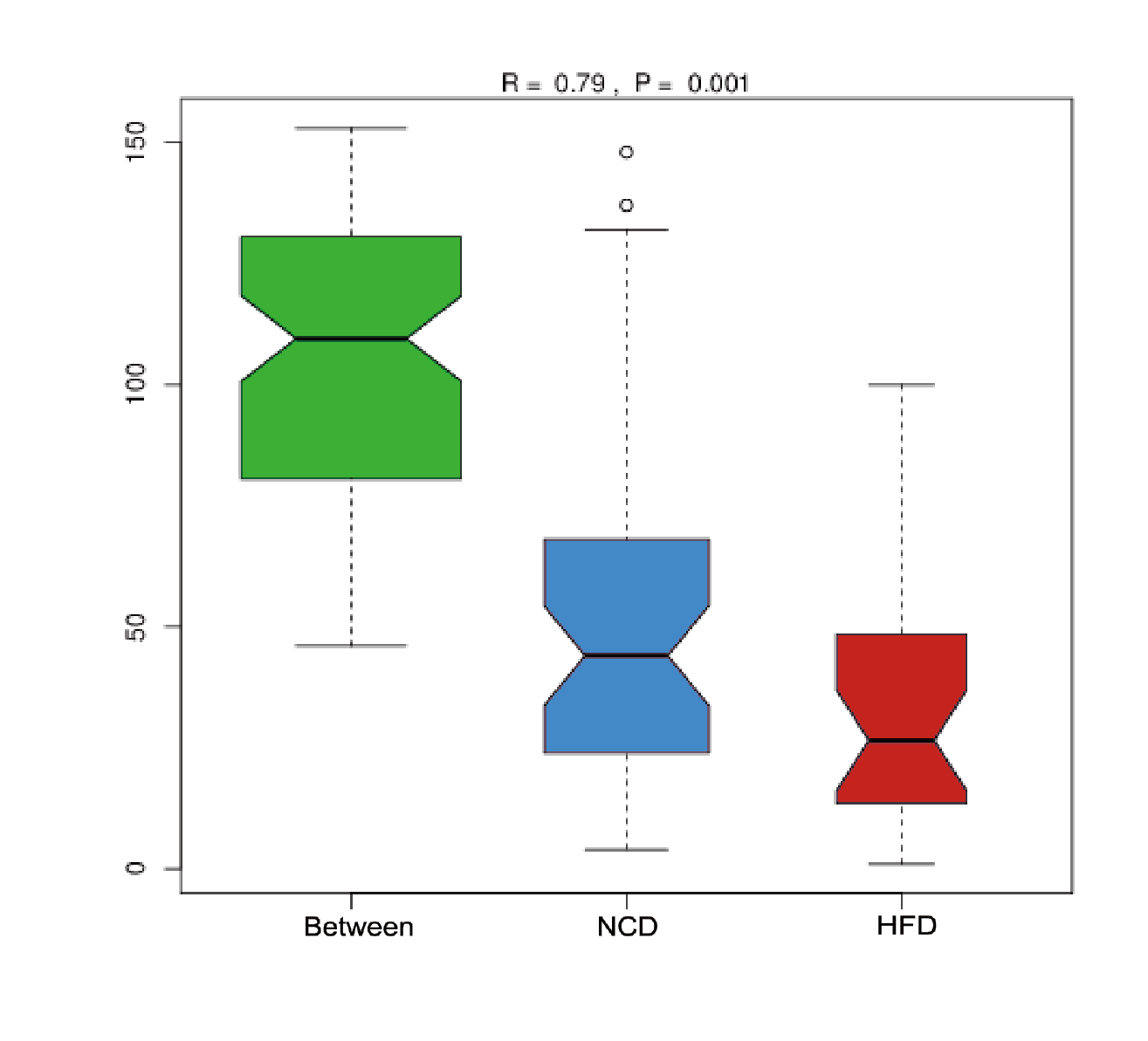

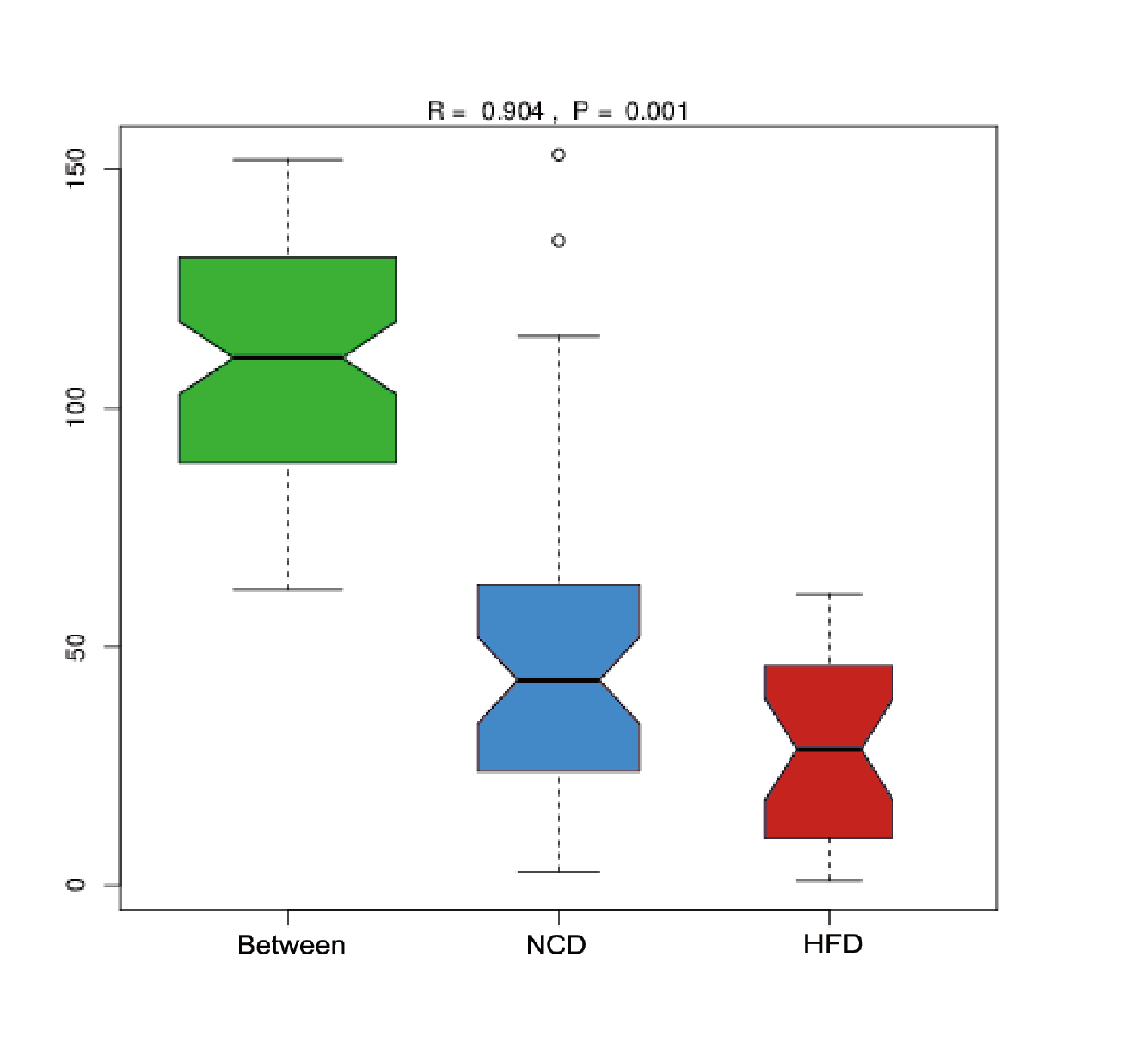
**

**Supplemental figure 2.** Analysis of microbial similarity based on ANOSIM at the genus and species levels

(**A**) analysis of microbial similarity at the genus level; (**B**) analysis of microbial similarity at the species level


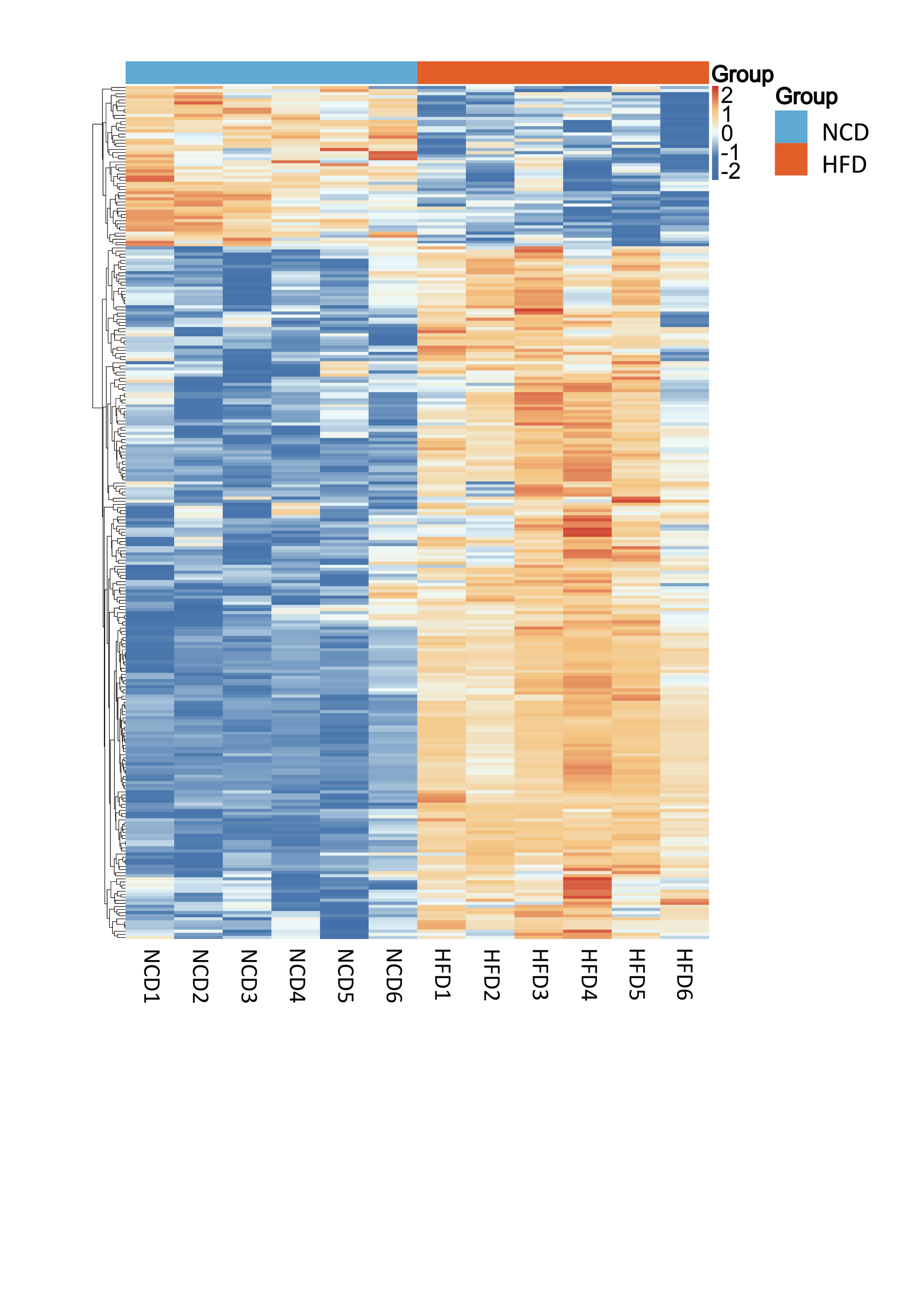


**Supplemental figure 3.** All differentially changed metabolites
